# High Dietary Folate in Mice Alters Immune Response and Reduces Survival after Malarial Infection

**DOI:** 10.1371/journal.pone.0143738

**Published:** 2015-11-24

**Authors:** Danielle N. Meadows, Renata H. Bahous, Ana F. Best, Rima Rozen

**Affiliations:** 1 Department of Human Genetics, McGill University, McGill University Health Center, Montreal, Quebec, Canada; 2 Department of Mathematics and Statistics, McGill University, Montreal, Quebec, Canada; 3 Department of Pediatrics, McGill University, McGill University Health Center, Montreal, Quebec, Canada; Oswaldo Cruz Institute (IOC-Fiocruz), BRAZIL

## Abstract

Malaria is a significant global health issue, with nearly 200 million cases in 2013 alone. Parasites obtain folate from the host or synthesize it *de novo*. Folate consumption has increased in many populations, prompting concerns regarding potential deleterious consequences of higher intake. The impact of high dietary folate on the host’s immune function and response to malaria has not been examined. Our goal was to determine whether high dietary folate would affect response to malarial infection in a murine model of cerebral malaria. Mice were fed control diets (CD, recommended folate level for rodents) or folic acid-supplemented diets (FASD, 10x recommended level) for 5 weeks before infection with *Plasmodium berghei* ANKA. Survival, parasitemia, numbers of immune cells and other infection parameters were assessed. FASD mice had reduced survival (p<0.01, Cox proportional hazards) and higher parasitemia (p< 0.01, joint model of parasitemia and survival) compared with CD mice. FASD mice had lower numbers of splenocytes, total T cells, and lower numbers of specific T and NK cell sub-populations, compared with CD mice (p<0.05, linear mixed effects). Increased brain TNFα immunoreactive protein (p<0.01, t-test) and increased liver *Abca1* mRNA (p<0.01, t-test), a modulator of TNFα, were observed in FASD mice; these variables correlated positively (r_s_ = 0.63, p = 0.01). *Bcl-xl/Bak* mRNA was increased in liver of FASD mice (p<0.01, t-test), suggesting reduced apoptotic potential. We conclude that high dietary folate increases parasite replication, disturbs the immune response and reduces resistance to malaria in mice. These findings have relevance for malaria-endemic regions, when considering anti-folate anti-malarials, food fortification or vitamin supplementation programs.

## Introduction

In 2013, there were 198 million reported cases of malaria in 97 malaria-endemic countries, with 584,000 deaths [1]. Malaria is caused by infection with the *Plasmodium* parasite and spreads through *Anopheles* mosquitos. In humans, the majority of morbidity and mortality comes from *Plasmodium falciparum* infection. Children, pregnant women, and poverty-stricken individuals are at greatest risk for poor outcomes [1, 2]. Typical features include flu-like signs such as fever, body aches, fatigue, and dehydration. In more complicated cases, severe anemia, splenomegaly, cerebral pathology and death may occur.

In mice, several strains of *Plasmodium* are used to study different complications of malaria. We have used *Plasmodium berghei* ANKA, a strain that induces experimental cerebral malaria in mice. We recently showed that a genetic deficiency in methylenetetrahydrofolate reductase (MTHFR, EC 1.5.1.20), an important enzyme in folate metabolism, protects against *P*. *berghei* ANKA infection [3]. *Mthfr* ^*+/-*^ mice, heterozygous for a null allele, were more resistant to *P*. *berghei* ANKA infection and mice that overexpressed MTHFR (through an additional copy of the human *MTHFR* gene (*MTHFR* ^*Tg*^ mice) were more susceptible to infection, compared with wild-type animals (*Mthfr* ^*+/+*^ mice); all mice had been fed standard mouse chow [3]. We found that resistant *Mthfr* ^*+/-*^ mice had differences in immune cell populations and cytokine expression, and that these changes could contribute to the increased survival [3].

Folate derivatives are required for nucleotide and amino acid synthesis, as well as for methylation reactions. For many years, anti-folate drugs have been used to inhibit growth of pathogens, including the malarial parasite. Two folate-dependent enzymes, dihydrofolate reductase (DHFR) and dihydropteroate synthase (DHSP) are popular targets in malaria [4, 5]. Blocking these enzymes inhibits *de novo* folate synthesis in the parasite; mammalian hosts do not synthesize folate. Unfortunately, resistance has developed to many of these drugs [4]. To limit the impact of disease, one preventative strategy has been the prescription of folate-blocking anti- malarials to late-term pregnant women and to children during routine health care screenings [1, 2]. The latest reports show that these programs are not reaching the desired level of efficacy and dissemination.

Low folate intake is a risk factor for neural tube defects. Consequently many countries have introduced folate fortification of food to reduce incidence [6–8]. The daily recommended amount of folate intake ranges from 0.2mg—0.4mg [9], with most countries adhering to the WHO’s recommendation of 0.4mg daily [10]. Higher doses, such as 4–5 mg (approximately 10-fold higher than the daily requirement) are sometimes recommended for women with a high-risk pregnancy [11, 12]. However, with fortification and the general increased use of vitamin supplements among many population sub-groups, there are concerns that increased folate intake may have negative health consequences, including immune cell cytotoxicity [13] and increased inflammation [14]. Food fortification and vitamin supplementation use folic acid, a synthetic folate that may inhibit folate-dependent enzymes and transporters [15]. These issues and others have collectively led to questions regarding the risks and benefits of universal fortification [6, 15]. Some malaria-endemic countries such as Ghana and Cote d’Ivoire have already implemented dietary fortification programs [16].

Our laboratory has shown that high folate intake in mice, using a diet with 10-fold higher folate than the recommended level for rodents [17], negatively impacts embryonic development [18] and leads to liver injury through alterations in lipid metabolism [19]. The higher dietary folate in these studies resulted in a 3-fold increase in plasma folate [18], the same degree of increase that has been reported in the United States [20]. Considering our finding that a genetic disturbance in folate metabolism has an impact on resistance to malaria and the fact that anti-folate drugs are frequently used as anti-malarials, we sought to determine whether increased dietary folate could affect the outcome of malaria infection in mice. We observed reduced resistance to malaria infection in mice with higher dietary folate, and identified some host changes in immune cell populations and immune modulators that could contribute to the altered resistance.

## Materials and Methods

### Ethics statement

All animal use protocols were in accordance with guidelines of the Canadian Council on Animal Care and were approved by the Montreal Children’s Hospital Animal Care Committee (Protocol #3132).

### Animals and diets

Male *Mthfr* ^+/+^ and *Mthfr* ^+/-^ mice [21] on a C57BL/6 background were housed in clean facilities with 12 hours light/dark cycles and fed *ad libitum*. *Mthfr* genotypes were determined by PCR [21] prior to infection and confirmed after death.

Five weeks prior to infection, mice with mean ages of 3–5 months were randomly assigned to groups fed one of the folate-defined diets: 1) CD + SST, an amino acid-based Control Diet (CD) that contained succinylsulfathiazole (SST), an antibiotic that prevents folate synthesis by intestinal flora. This diet contains the recommended amount of folic acid for rodents (2mg/kg diet) [17] (TD.090704 Folic Acid Control Diet, a modified version of TD01369 [22], Harlan Laboratories, Inc., Madison, WI, USA); 2) CD, the same Control Diet without SST (TD.130565 Folic Acid Control Diet, Harlan Laboratories, Inc., Madison, WI, USA); 3) Folic Acid-Supplemented Diet (FASD), a diet identical to CD without SST except that it is supplemented with folic acid at 10 times the amount in the CD diet (20 mg folic acid/kg diet, TD.130998, a modified version of TD.09258 [18], Harlan Laboratories, Inc., Madison, WI, USA). All diets had their vitamin levels (including folic acid) adjusted to allow for irradiation (as recommended by the supplier), so that vitamin levels were at the desired levels post-irradiation. In experiments comparing FASD to CD, a few mice were fed mouse chow as positive controls to confirm the viability and virulence of the parasite inoculums. The mouse chow (2920x Irradiated Teklad Global Soy Protein-Free Extruded Rodent Diet, Harlan Laboratories Inc., Madison, WI, USA) contains a mix of whole food products and is estimated to contain approximately 4 mg folic acid /kg diet as well as other sources of one-carbon donors (methionine, choline, and serine).

### *Plasmodium berghei* ANKA Infection

After 5 weeks on diets, mice were randomly administered an inoculum of 200μl phosphate-buffered saline with 10^5^ parasite-infected red blood cells (pRBCs), delivered by intravenous tail injection. Parasites were maintained through infection of naïve mice and inoculums prepared fresh on the day of infection. The parasite dose was determined as previously described [3]. Animals were closely monitored for survival after infection or were euthanized at the predefined endpoint as previously reported [3].

### Measurement of blood parasites

Starting at 6 days post-infection (dpi) and collecting on alternate days, blood was collected from the tails and smeared in a thin layer on microscope slides. RBC slides were stained with the Hemacolor kit (Harleco, EMD Millipore, USA). Parasitemia was determined by calculating the ratio of pRBCs to uninfected RBCs.

### Tissue collection

At 7 dpi and in random order, mice were asphyxiated in a CO_2_ chamber. Blood was collected via cardiac puncture, and thin-layer smears were generated. Brain, liver, and spleen were harvested by dissection and tissues were frozen at -70°C until use.

### Assessment of immune cell populations

At 7 dpi, animals were sacrificed and spleens harvested. Splenocytes were prepared as previously described [3] and then seeded at 5.0x10^6^ cells for incubation with GolgiStop (BD Biosciences, Mississauga, ON, Canada) for 3 hours. After incubation, 1x10^6^ cells were stained for viability with Live/Dead Fixable Aqua Stain (LifeTechnologies, Burlington, ON, Canada). After washing, cells were stained with a mix of surface antibodies: APC-eFluor 780 anti-CD4 (eBioscience, San Diego, CA, USA), FITC anti-DX5 (BioLegend, San Diego, CA, USA), PE anti-CD194/chemokine receptor 4 (CCR4) (BioLegend, San Diego, CA, USA), APC anti-CD183/CXCR3 (BioLegend, San Diego, CA, USA), and eFlour450 anti-CD8 (eBioscience, San Diego, CA, USA). Cells were fixed and permeabilized (BD Cytofix/ Cytoperm Fixation and Permeablization Kit, BD Biosciences, Mississauga, ON, Canada). After permeabilization, cells were intracellularly stained with a mix of antibodies: PE/Cy7 anti-interferon gamma (IFNγ) (BioLegend, San Diego, CA, USA) and PerCP-eFluro710 anti-CD3 (eBioscience, San Diego, CA, USA). Samples were run on a BD Canto Flow Cytometer (BD Biosciences, Mississauga, ON, Canada) and 2.0x10^5^ events were recorded. Data were analyzed with FlowJo software (Treestar Inc, Ashland, OR, USA).

### Immunoblotting for brain tumor necrosis factor α (TNFα)

Preparation of protein lysates for electrophoresis and immunoblotting were performed as previously described [23]. Primary antibodies for TNFα (Abcam, Cambridge, MA, USA), and β-actin (Sigma-Aldrich, Oakville, ON, Canada) were used with the peroxidase-coupled anti-rabbit IgG secondary antibody (Amersham, GE Healthcare Live Sciences, Piscataway, NJ, USA). Signal was detected using the ECL Plus chemiluminescence system (Amersham, GE Healthcare Life Sciences, Piscataway, NY, USA) and visualized with film. Densitometry was performed using Quantity One 4.1.0 software (BioRad Laboratories, Mississauga, ON, Canada) and protein quantified using β-actin as an internal control. CD mice were used to generate a mean value that was designated as 1 and the FASD mice were calculated as a ratio of the CD value for each blot.

### RNA extraction and quantitative reverse-transcriptase-PCR

RNA was extracted from frozen liver (~15mg) using the RNeasy Tissue Mini Kit (Qiagen, Toronto, ON, Canada) according to manufacturer’s instructions. Reverse transcription was performed as previously described [24]. Quantitative real-time PCR reactions were performed using Platinum SYBR Green master mix (Invitrogen, Burlington, ON, Canada), with primers described in Table 1, using the Roche Lightcycler 480 II (Roche Life Science, Laval, QC, Canada). Primers were designed using NCBI PrimerBLAST [25]. Expression of target genes was normalized to glyceraldehyde-3-phosphate dehydrogenase (*Gapdh)*, and tyrosine 3-monooxygenase/tryptophan 5-monooxygenase activation protein zeta (*Ywhaz)*, using a normalization factor calculated by geNorm v.3.4 [26].

**Table 1:** Primers for quantitative real-time RT-PCR.

Gene	Direction	Primer Sequence	Amplicon Size	T_m_ (°C)	Reference
*Gapdh*	Forward	CAGGAGCGAGACCCCACTAACAT	74	62	[27]
	Reverse	AAGACACCAGTAGACTCCACGAC			
*Ywhaz*	Forward	TGCTGGTGATGACAAGAAAGGA	119	60	[28]
	Reverse	TGAGGGCCAGACCCAGTCT			
*Bak*	Forward	TATTAACCGGCGCTACGACAC	109	60	[28]
	Reverse	CTTAAATAGGCTGGAGGCGATCTT			
*Bcl-xl*	Forward	GGTAGTGAATGAACTCTTTCGGGAT	131	60	[28]
	Reverse	TCCGACTCACCAATACCTGCAT			
*Abca1*	Forward	CGTTTCCGGGAAGTGTCCTA	78	60	[29]
	Reverse	CTAGAGATGACAAGGAGGATGGA			

The table includes expected amplicon size, melting temperature (T_m_) and reference source. Housekeeping genes (*Gapdh* and *Ywhaz*) were used to generate the normalization factor.

### Statistical analyses

Analyses of survival, parasitemia, and immune cell counts were performed using R version 3.2.0 [30]. The survival analysis was performed using the survival package [31, 32].Two experiments were combined directly to produce a joint Kaplan-Meier plot [33], and combined using frailty methods in a Cox proportional hazards model [34] to account for within-experiment clustering. Additionally, log-rank tests accounting for frailty [35] were performed. Parasitemia was analyzed using the JM [36] package in order to jointly model survival and the longitudinal parasitemia observations, since deaths are informative with regards to parasitemia. For the longitudinal portion of the joint model, a linear mixed effects model was used; the natural log of parasitemia was chosen as the response, with fixed effects of time, diet, genotype, and the diet and genotype interactions with time, and a random effect of mouse ID. For the survival portion of the joint model, the baseline hazard of death was modeled using B-splines, and diet and genotype effects were modeled using Cox’s proportional hazards model with frailty. All observed parasitemia values of 0 were imputed to 0.1%, the smallest nonzero observable value; a sensitivity analysis was carried out to determine the effect of this imputation, which was determined to be minimal. The imputation of all parasitemia values below the detection threshold to a single value violates the normality assumptions of the linear mixed effects model; however, these values comprise a small proportion of the available data and diagnostic plots indicated no other departures from the model assumptions. The immune cell counts were analyzed using the nlme [37] package as linear mixed effects, with experiment as a random effect and diet and genotype as fixed effects

Correlations and unpaired t-tests were performed using GraphPad v6 (GraphPad Software Inc, La Jolla, CA, USA). Welch’s correction was applied to adjust t-tests for unequal variances. When combining ratio expression data, CD mice in each experiment were standardized to a mean expression level or intensity ratio of 1, and the values for FASD mice were calculated as a proportion of the value for CD mice. In comparative graphs, data are presented as mean ± standard error of the mean (SEM).

## Results

### Decreased survival of infected mice fed FASD

**Fig 1:**
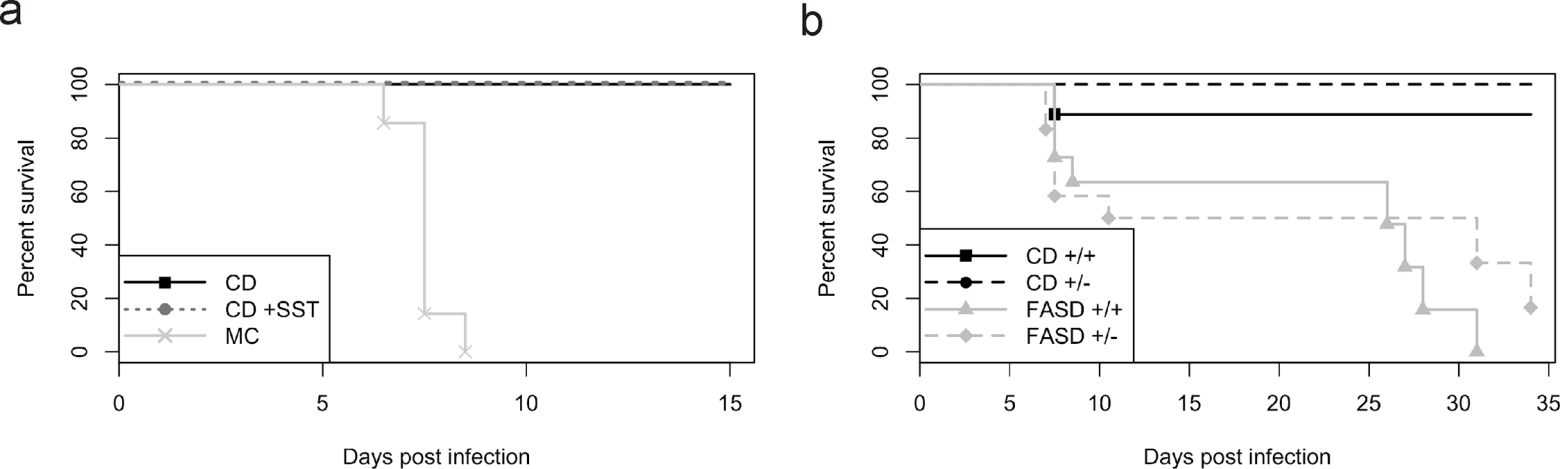
Survival of mice fed diets with variable folate content. (a) CD mice (n = 8) and CD+SST mice (n = 7) did not show differences in survival at 15 dpi (p = 1.0, log-rank test); MC mice (n = 7) had 100% mortality by 10 dpi. (b) Survival of CD^+/+^ mice (n = 9), CD^+/-^ mice (n = 14), FASD^+/+^ mice (n = 11), and FASD^+/-^ mice (n = 12) in two experiments combined. Mice fed CD had a greater chance of surviving infection than mice fed FASD (p<0.01, Cox proportional hazards). *Mthfr* genotype had no effect on chance of survival in mice fed CD or FASD (p = 0.45, Cox proportional hazards).

Murine diets that investigate the impact of folate intake, as used in previous work in our laboratory, often contain an antibiotic to prevent folate synthesis by intestinal flora [38]. To ensure that the antibiotic routinely used in our control diet, succinylsulfathiazole (SST), did not affect survival of mice infected with the malarial parasite, we compared survival of male *Mthfr* ^*+/+*^mice fed the control diet with SST (CD+ SST) to that of male *Mthfr* ^*+/+*^mice fed the control diet without SST (CD), and included mice fed mouse chow (MC) as a positive control. Both control diets are amino acid defined and contain the recommended amount of folic acid for rodents (2 mg/kg diet), while the mouse chow is made up of whole food products and is estimated to contain 4 mg/kg diet). After 5 weeks on diet, *Mthfr* ^*+/+*^ mice were injected with 10^5^ *P*. *berghei* ANKA parasites and survival was monitored. There was 100% survival in both CD groups at two weeks post-infection. Survival between CD and CD+SST mice did not differ (Fig 1A; p = 1.0, log-rank test). Onset of mortality was observed in MC mice at 7 or 8 dpi, with 100% mortality before 10 dpi. This result is the typical outcome in mice fed standard chow and dying of malaria, as observed by our group [3] and others [39, 40].

Mouse chow and amino acid-defined diets, such as CD and CD+SST, are very different in content, not just in the level of folic acid, but also in other amino acids and nutrients, including methionine and serine which can participate in one-carbon metabolism. With so many other methyl donors in the diet at such variable levels, it would be impossible to address our main question of whether the outcome of malaria infection could be influenced by the level of folic acid in the diet.

To determine whether differences in survival were due only to differences in folate content, we used a folic acid- supplemented diet (FASD), in which the diet was identical to CD in all components except for a 10-fold increase in folate content (20 mg/kg diet). Since there were similar rates of survival between CD and CD+SST mice, we did not add SST to our CD or FASD diets. After five weeks on CD or FASD, *Mthfr* ^*+/+*^ (CD^*+/+*^ and FASD^+/+^) and *Mthfr* ^*+/-*^ (CD^*+/-*^ and FASD^+/-^) mice were infected with 10^5^ *P*. *berghei* ANKA parasites and survival was monitored.

The survival analyses combined two experiments, one of which was terminated at 20 days while the other was terminated at 34 days. The Kaplan-Meier curves for the four genotype/diet groups are depicted in Fig 1B. There was no evidence of a statistically significant difference in survival between *Mthfr* ^*+/+*^ and *Mthfr* ^*+/-*^ mice fed CD (n = 9 and n = 14 respectively, p = 0.08, log rank test with frailty) or *Mthfr* ^*+/+*^ and *Mthfr* ^*+/-*^ fed FASD (n = 11 and n = 12 respectively, p = 0.13, log rank test with frailty).

Fig 1B indicates that CD mice survived significantly longer than FASD mice (p<0.01, Cox proportional hazards). In the presence of diet, there was no evidence of a statistically significant effect of genotype on survival (p = 0.45, Cox proportional hazards) or of an interaction between diet and genotype (p = 0.45, analysis of deviance). These results were confirmed by log-rank tests with frailty, and by the survival component of our joint modeling of parasitemia (discussed below). Neurological signs consistent with cerebral malaria such as tremors, lethargy, impaired gait, and mortality were observed in some of the FASD mice starting at 8–9 dpi. It is unclear whether the ultimate demise after 20 dpi was due to cerebral complications or from some other complications of severe malaria, such as severe anemia.

### Increased parasite levels in infected FASD mice

**Fig 2:**
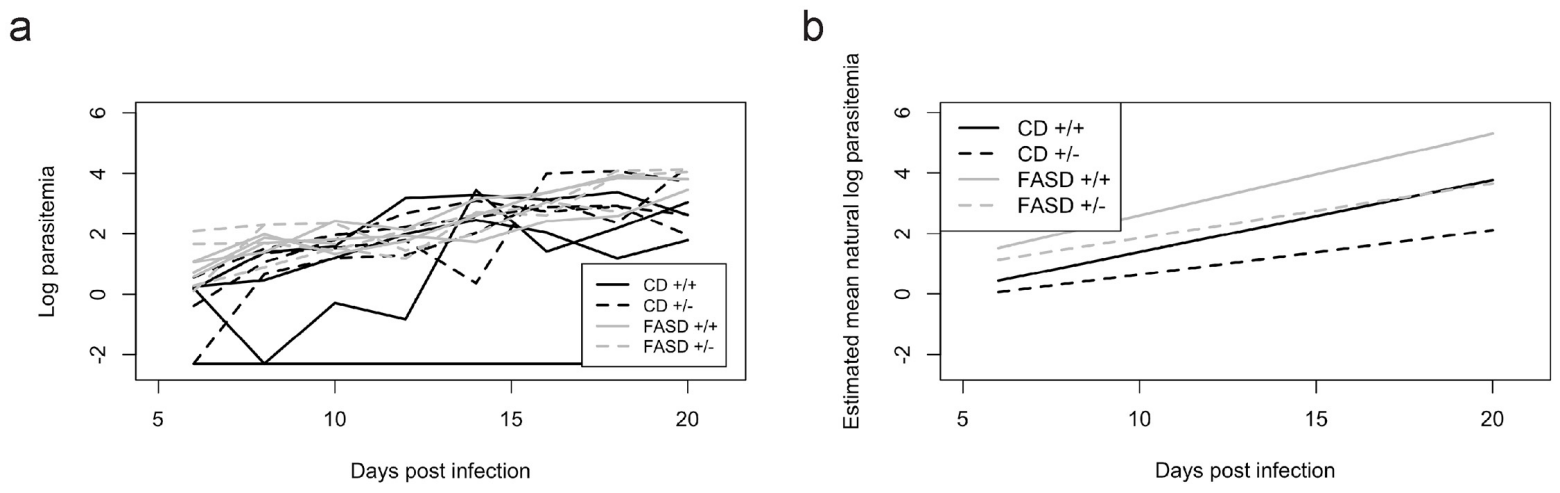
Parasitemia in CD and FASD mice. Parasitemia was measured every 48h from 6–20dpi. (a) Observed parasitemia trajectories from 5 randomly selected CD^*+/+*^ (n = 9), CD^*+/-*^ (n = 14), FASD^*+/+*^ (n = 11), and FASD^*+/-*^ (n = 12) mice from two combined experiments; selected trajectories are representative of the full data. (b) Statistical model showing estimated mean natural log of the parasitemia used to interpret parasitemia. The FASD mice had significantly higher overall levels of parasitemia than CD mice (p< 0.01, joint model of parasitemia and survival).

Parasitemia was measured every 48h from 6–20 dpi (Fig 2) during survival experiments. Fig 2A plots the observed natural log parasitemia trajectories for 5 randomly selected mice from each diet/genotype group; all mice, however, were used for analysis. Color banding indicates, informally, that FASD mice experienced higher parasitemia than CD mice. The estimated curves in Fig 2B plot the estimated mean natural log of parasitemia as a function of time for the four diet/genotype groups. These estimated curves result from our joint model of parasitemia and survival, necessary due to the significant dropout of dying FASD mice.

Parasitemia was higher overall in FASD mice than CD mice (p<0.01, joint model of parasitemia and survival). There was a borderline statistically significant increase in the rate of parasitemia in FASD mice relative to CD mice (p = 0.08, joint model). There was no evidence of an overall effect of genotype on parasitemia (p = 0.58, joint model), but parasitemia increased more rapidly in *Mthfr* ^*+/+*^ mice than *Mthfr* ^*+/-*^ mice (p<0.0001, joint model).

### Decreased immune cell populations in infected FASD mice

**Fig 3:**
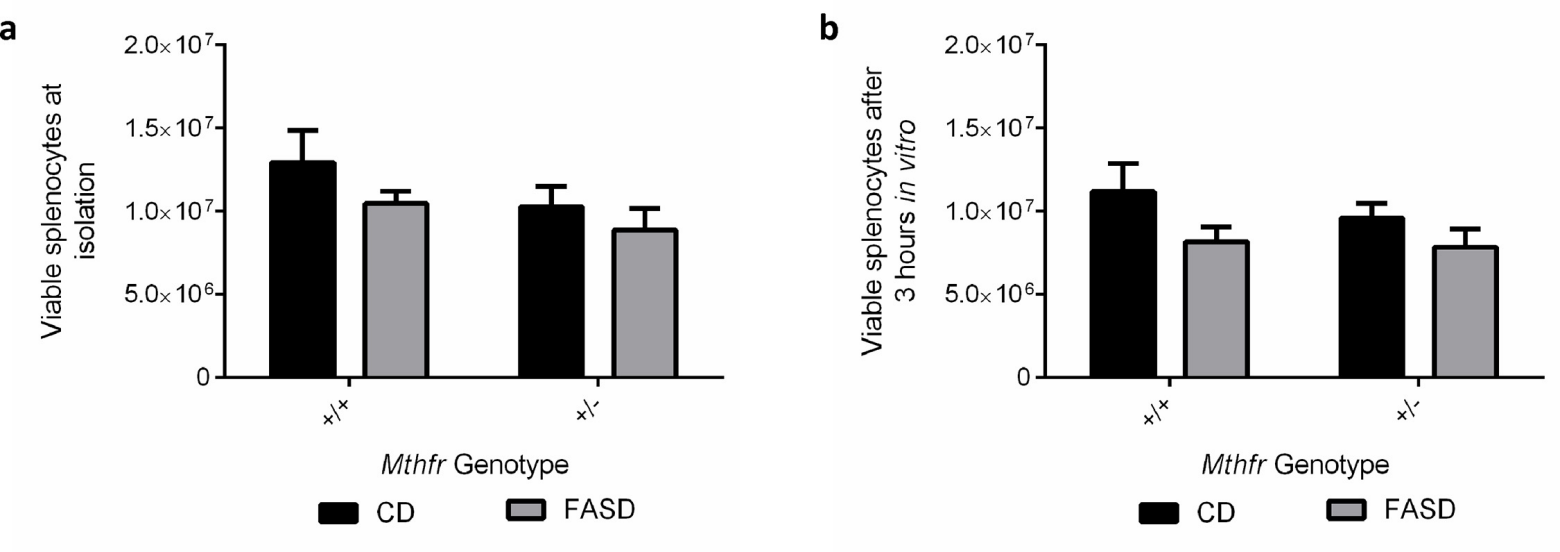
Differences in viable immune cell populations at 7 dpi between CD and FASD mice. Values are means ± SEM. Two experiments are combined for visual simplicity, but separated in the analysis using a linear mixed effects model. (a) FASD mice (grey; n = 8 (*Mthfr* ^*+/+*^) and n = 6 (*Mthfr* ^*+/-*^)) had lower numbers of viable splenocytes compared with CD mice (black; n = 8 (*Mthfr* ^*+/+*^) and n = 7 (*Mthfr* ^*+/+*^)) (p<0.05, linear mixed effects). (b) Viable splenocyte numbers were lower in FASD murine cultures after incubation with GolgiStop™ (p = 0.05, linear mixed effects).

In earlier work [3], we found increases at 6 dpi in some immune cell populations in the spleen of mice that were resistant to *P*. *berghei* ANKA. To examine the same cell populations in this study, we isolated splenocytes at a similar time point. Splenocytes were collected at 7 dpi, just before the onset of neurological signs or deaths in FASD mice at 8–9 dpi, as this time point might provide some information regarding an initial immune response. We isolated splenocytes from CD and FASD mice at 7 dpi, counted viable cells and performed flow cytometry. Two independent experiments were performed and combined for analysis. FASD mice had significantly less viable splenocytes at isolation compared with CD mice (Fig 3A; p<0.05, linear mixed effects). Viable splenocytes were then plated at equal densities (5.0 x 10^6^ cells) and incubated at 37°C with cell media enriched with the cytokine secretion inhibitor GolgiStop After 3 hours in culture, cells were re-stained for viability. Again, FASD mice had less viable cells than CD mice (Fig 3B; p = 0.05, linear mixed effects), despite being plated at equal densities of viable cells.

**Fig 4:**
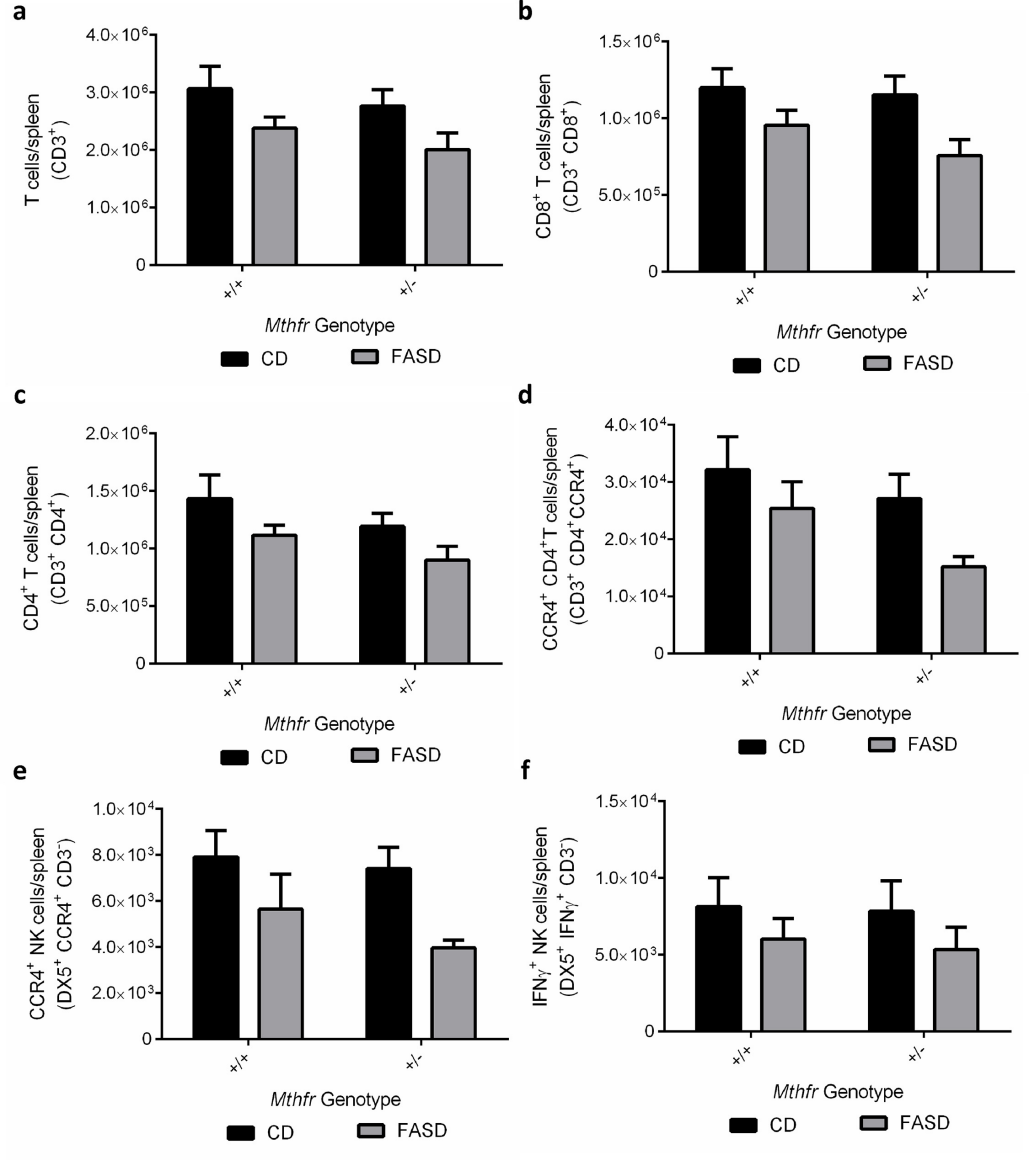
Differences in spleen immune cell populations at 7 dpi between CD and FASD mice. Values are means ± SEM. Two experiments are shown. FASD mice (grey; n = 8 (*Mthfr* ^*+/+*^) and n = 6 (*Mthfr* ^*+/-*^))had significantly decreased populations of (a) T cells (p = 0.01, linear mixed effects), (b) CD8^+^ T cells (p<0.01, linear mixed effects), (c) CD4^+^ T cells (p<0.05, linear mixed effects), (d) CCR4^+^ CD4^+^ T cells (p<0.05, linear mixed effects), (e) CCR4^+^ NK cells (p<0.01, linear mixed effects) and (f) IFNγ^+^ NK cells (p<0.05, linear mixed effects) compared with CD mice (black; n = 8 (*Mthfr* ^*+/+*^) and n = 8 (*Mthfr* ^*+/+*^)).

Staining for surface cellular differentiation markers CD4, CD8, DX5 (CD49B), CCR4 (CD194) and intracellular staining for CD3 and IFNγ allowed us to identify and quantify various immune cell populations. Our staining panel specifically targeted natural killer (NK) and T cell populations, which are critical in fighting malarial infection [41, 42]. Shown are the cell populations that had significant differences between CD and FASD mice (Fig 4A–4F).

FASD mice had decreased populations of T cells (Fig 4A; p = 0.01, linear mixed effects). Within T cell subsets, they had decreased CD8^+^ T cells (Fig 4B; p<0.01, linear mixed effects), as well as decreased populations of CD4^+^ T cells (Fig 4C; p<0.05, linear mixed effects). Within the CD4^+^ T cell population, FASD mice also had decreased CCR4^+^ CD4^+^ T cells (Fig 4D; p<0.05, linear mixed effects).

There were no changes in the total number of NK cells, but FASD mice had fewer CCR4^+^ NK cells (Fig 4E; p<0.01, linear mixed effects) than CD mice. They also had a decrease in IFNγ^+^ NK cells (Fig 4F; p<0.05, linear mixed effects). CCR4^+^ NK cells have been reported to produce IFNγ and IL-17 [43], which are important responders in malarial infection.

There were no differences due to genotype in the numbers of immune cells except for a small but significant effect on the CD4^+^ T cell population, with *Mthfr* ^*+/+*^having slightly larger populations of CD4^+^ T cells compared with *Mthfr* ^*+/-*^ littermates (Fig 4C; p<0.05, linear mixed effects).

### Increased expression of TNFα in brain and increased *Abca1* mRNA in liver of FASD mice

**Fig 5:**
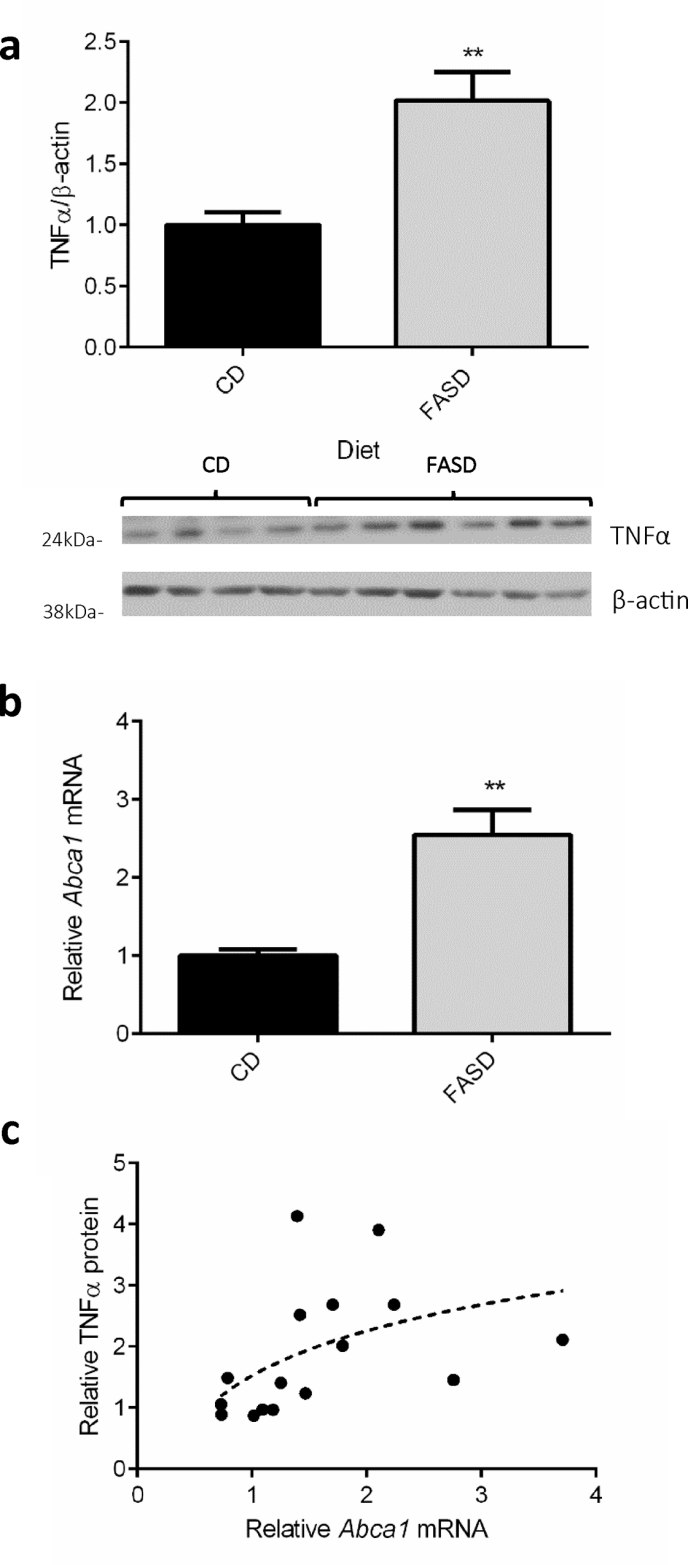
Differences in TNFα immunoreactive protein in brain and in relative *Abca1* mRNA in liver between CD and FASD *Mthfr* ^*+/+*^ mice at 7 dpi. Values are means ± SEM for A and B. (a) FASD mice (grey bar; 3 experiments combined; n = 10) had significantly higher levels of TNFα protein in brain compared with CD mice (black bar; 3 experiments combined; n = 7). Representative Western blot is shown below the graph. (b) Livers of FASD mice (grey bar; 3 experiments combined; n = 9) had significantly higher relative *Abca1* mRNA levels than livers of CD mice (black bar; 3 experiments combined; n = 9)). (c) Brain TNFα protein correlated with *Abca1* mRNA levels in liver of mice in both dietary groups (r_s_ = 0.63, Spearman correlation, p = 0.01; n = 16). **p<0.01, unpaired t-test.

Severe malaria outcomes are due to an exacerbated inflammatory response to fight the infection [44]. One of the primary mediators of this inflammatory response is TNFα; decreases in TNFα have been consistently shown to improve the outcome of cerebral malaria [45–47]. We measured the expression in brain of TNFα (Fig 5A) by immunoblotting, in *Mthfr* ^*+/+*^ animals fed CD or FASD in 3 different experiments. FASD mice had an approximate 2-fold increase in TNFα protein, compared with CD mice (Fig 5A; p<0.01, t-test with Welch’s correction).

High levels of TNFα may relate, at least in part, to increased expression of ATP-binding cassette sub-family A, member 1, *Abca1*, a gene important in cholesterol transport. *Abca1* ^-/-^ mice have fewer pro-inflammatory microparticles in plasma and, decreased brain TNFα compared with wild-type littermates, and are protected from cerebral malaria. These observations in *Abca1* ^-/-^ mice suggested that brain TNFα expression may be regulated by *Abca1* expression [48]. We therefore elected to measure *Abca1* mRNA in liver, as *Abca1* expression is quite high in this tissue. We found that FASD mice had an approximate 2-fold higher level of *Abca1* expression compared with CD mice (Fig 5B; p<0.01, t-test with Welch’s correction). Brain TNFα expression levels positively correlated with liver *Abca1* expression levels in CD and FASD mice (Fig 5C; r_s_ = 0.63, Spearman correlation, p = 0.01).

### *Bcl-xl/Bak* ratio suggests reduced apoptotic environment in liver of FASD mice

Liver pathology, driven by resident parasites, often precedes cerebral pathology in *P*. *berghei* ANKA infection, and removal of parasites can reverse the liver pathology [49]. To explore liver apoptotic potential, we measured mRNA levels of B cell lymphoma-extra-large *(Bcl-xl)* and BCL2-agonist/killer 1 *(Bak)*, and determined their ratio in CD and FASD *(Mthfr* ^*+/+*^ *)* mice (Table 2). *Bcl-xl* mRNA levels were increased in FASD mice (p<0.05, t-test with Welch’s correction), with no differences in *Bak* mRNA between dietary groups. In FASD mice, the *Bcl-xl/Bak* ratio was higher compared with that in CD mice (p<0.01, unpaired t-test with Welch’s correction). This higher ratio is suggestive of a reduced apoptotic environment [28, 50] in FASD mice.

**Table 2:** Relative *Bcl-xl*, *Bak*, and *Bcl-xl/Bak* mRNA in liver of CD and FASD *(Mthfr* ^*+/+*^ *)* mice at 7 dpi.

	mRNA expression		
	CD	FASD	
*Bcl-xl*	1.00 ± 0.09	1.68 ± 0.25	p<0.05
*Bak*	1.00 ± 0.07	1.02 ± 0.13	p>0.05
*Bcl-xl/Bak*	1.00 ± 0.07	1.71 ± 0.163	p<0.01

Expression of *Bcl-xl*, *Bak*, and *Bcl-xl/Bak* in liver of CD mice (n = 9; 3 combined experiments) and FASD mice (n = 9; 3 combined experiments) indicates that FASD murine livers had a lower apoptotic environment (higher *Bcl-xl/Bak* ratio) compared with that in CD mice.

## Discussion

Folate is required for synthesis of nucleotides and amino acids, and for methylation reactions. The malarial parasite also requires folate for these functions, and obtains folate derivatives from the host, or, unlike the host, can synthesize folate *de novo* [4, 5, 51]. Drugs that target folate metabolism in the parasite have been in use for many years, and reduce the availability of important building blocks for parasite replication.

Our work indicates that restriction of dietary folate results in reduced parasitemia and increased survival, as observed in mice fed CD. Our control diet has the recommended amount of folate necessary for normal function of the host, but it has less folate than the standard laboratory mouse chow routinely used for experimental malarial studies, and less folate than FASD. In addition to higher folate content, mouse chow also has higher levels of other one-carbon donors, such as methionine and serine, which can be metabolized for one-carbon transfer reactions. Our findings are consistent with those of an earlier study where lack of p-aminobenzoic acid (pABA), a folate precursor, in the diet led to lack of growth of *Plasmodium yoelii* and increased survival of the host [52]. In our experiments, it appears that the effect of dietary folate restriction is much more profound than the effect of the genetic *Mthfr* deficiency [3]. This observation, i.e. a greater effect of diet than genotype, is similar to that in our previous work on the effect of *Mthfr* deficiency and dietary folate on tumor development [53, 54] or pregnancy outcomes [18, 55]. It may reflect the fact that reducing the total folate pool (as with the CD diet) is more critical than decreased availability of a particular folate derivative. Although *Mthfr*-deficient mice showed significant differences in survival from that seen in wild-type animals, these mice had been fed mouse chow with adequate total one-carbon donors and the differences in survival between the genotype groups were not as dramatic as the differences in survival between dietary groups in this report.

Increased blood folate has been observed in North America and other countries [56, 57]. Folic acid, a synthetic form of the vitamin used in food fortification and vitamin supplements, is included in the total folate pool and appears at greater levels in the circulation, compared to pre-fortification values [58]. Studies are emerging alluding to the potential negative consequences of high folate intake, although it is not clear whether the concerns relate to the increase in the total folate pool or to the unmetabolized folic acid. These concerns include immune dysfunction [13] and lower performance in cognitive tests [59, 60]. In other work using murine models, we demonstrated increased heart defects in embryos of dams fed high folate during pregnancy [18], and disturbances in lipid metabolism and liver injury in adult mice fed high folate diets for 6 months [19]. Folic acid may inhibit folate-dependent enzymes or transporters. In the liver study, we showed that folic acid can inhibit MTHFR activity but it also decreased MTHFR immunoreactive protein in liver of mice fed FASD [19]. In the current study, we have no evidence for a direct effect on MTHFR since our diets were administered for a short period of time.

The decreased resistance to malaria in mice fed FASD presumably relates to the increased availability of folate for growth of the parasite. Similar conclusions were reached in studies where therapeutic administration of folic acid tablets appeared to minimize the efficacy of anti-folate anti-malarial drugs [61, 62]. However, it is likely that changes in the host immune response also contribute to the outcome. In FASD mice, we observed lower levels of splenocytes, T cells and some sub-populations of T cells and NK cells, compared with levels in CD mice. These immune cells are usually up-regulated to fight malarial infection [41, 42]. Although the exact mechanisms responsible for the change in immune cells require identification, it is possible that folic acid inhibits other critical folate-dependent enzymes involved in proliferation, methylation or immune function. The decreases in some NK populations in FASD mice are also consistent with a report suggesting that high levels of unmetabolized folic acid may be toxic to human NK cells [13].

The observation of increased immune cells in the resistant CD mice, is consistent with the increased immune cells in resistant *Mthfr* ^*+/-*^ mice in our earlier report, and with the decrease in immune cells in the susceptible *MTHFR* ^*Tg*^ mice [3]. As we proposed in that study, the immune cells in blood may simply be “spill over” (reviewed by Tisoncik et al, [63]), and may not be indicative of immune cell numbers in the primary tissue (brain). The susceptible FASD mice may have more T cells recruited to brain and therefore fewer cells are detected in spleen. This hypothesis is supported by our finding of increased brain TNFα in FASD mice. These data may help to explain the occurrence of more severe and lethal disease early in the infection in FASD mice. Whether the early immune cell population changes contributed to the later deaths (after 25 dpi) is not known.

Cerebral malaria results from an exacerbated inflammatory response in the brain [44]. In our experiments, the FASD mice exhibited severe neurological signs including tremors and impaired motor function before death; these signs were not observed in CD mice. However, although these features are consistent with cerebral malaria, the diagnosis was not confirmed by pathology. We also observed increases in brain TNFα in FASD mice. Decreases in TNFα have been consistently shown to improve the outcome of cerebral malaria [45, 47, 64]. High levels of TNFα are present in early stages of infection and *in vivo* neutralization of TNFα can prevent symptoms [45]. The increase in the immunomodulator TNFα in FASD mice is consistent with the poorer outcome and the observed neurological signs. *Abca1* ^*-/-*^ mice survive cerebral malaria after *P*. *berghei* ANKA infection and have significantly lower levels of TNFα than wild-type mice. *Abca1* increases release of microparticles following malarial infection; these microparticles are involved in inflammation and result in an increase of TNFα [48]. Our finding of increased *Abca1* expression with increased TNFα expression in FASD mice, and the positive correlation between TNFα and *Abca1* in all mice, is consistent with a significant disturbance in the immune response of the host and with the poor outcomes attributed to an increase in TNFα in FASD mice.

The higher *Bcl-xl/*Bak ratio in liver of FASD mice is suggestive of a reduced apoptotic environment compared with that in liver of CD mice. Parasite clearance in liver protects against cerebral symptoms [65], and *P*. *berghei* ANKA typically blocks apoptosis in liver [66]. The altered hepatic environment with high dietary folate may represent another mechanism that enhances parasite expansion, in addition to a simple change in one-carbon availability for nucleotide and amino acid synthesis.

In the last few decades, there has been considerable effort to increase folate intake to minimize the incidence of neural tube defects and other disorders [15]. Food fortification with folate is routine in many countries worldwide, including some countries in malaria-endemic region [16]. Pregnant women, children and the elderly often use vitamin supplements that contain folic acid. Fortification and supplementation have led to increased blood folate [13, 58, 59]; increased blood folate has been suggested to limit the efficacy of anti-folate anti-malarial treatment [61, 62]. It is interesting to note that a study in Malawi found that children from a region with high vegetable intake had greater risk of malarial treatment failure than children from a region with a predominant fish or meat diet; the authors speculated that the higher folate in the vegetable diet could have contributed to this difference [67].

Although there is a body of literature suggesting the potential impact of folic acid on malarial infection, our work is novel in its direct demonstration of the impact of dietary folate on resistance to malarial infection and on the host response in relevant tissues. The finding that our control diet led to virtually complete resistance to malarial infection is particularly striking. This observation lends itself to new avenues for investigation of malarial resistance, particularly since there are limited studies examining the effects of single nutrients on malarial outcome; one report found partial protection with a vitamin E-deficient diet [68].

The increased intake of folate, prior to malarial infection, was associated with increased parasite levels in blood, decreased amounts of critical immune cells, and an increase in brain of a major cytokine, TNFα, which leads to the observed neurological signs. Increased dietary folate could lead to decreased resistance to malaria in human populations or to reduced efficacy of anti-folate drugs. Additional studies are warranted to examine the impact of variable dietary folate in human malaria, to ensure that excess folate, through fortification and/or vitamin supplementation, is not particularly harmful in malarial-endemic regions.
